# Fatores associados à piora no estilo de vida durante a pandemia de COVID-19 na população brasileira de lésbicas, gays, bissexuais, transexuais, travestis e identidades relacionadas: estudo transversal

**DOI:** 10.1590/S1679-49742022000100005

**Published:** 2022-02-25

**Authors:** Luciana Helena Reis Braga, Cynthia Santos Menezes, Isadora Viegas Martins, Janderson Diego Pimenta da Silva, Juliana Lustosa Torres

**Affiliations:** 1 Universidade Federal de Minas Gerais, Departamento de Gestão de Serviços de Saúde, Belo Horizonte, MG, Brasil Universidade Federal de Minas Gerais Universidade Federal de Minas Gerais Departamento de Gestão de Serviços de Saúde Belo Horizonte MG Brazil; 2 Universidade Federal de Minas Gerais, Departamento de Direito, Belo Horizonte, MG, Brasil Universidade Federal de Minas Gerais Universidade Federal de Minas Gerais Departamento de Direito Belo Horizonte MG Brazil; 3 Universidade do Estado de Mato Grosso, Departamento de Enfermagem, Tangará da Serra, MT, Brasil Universidade do Estado de Mato Grosso Universidade do Estado de Mato Grosso Departamento de Enfermagem Tangará da Serra MT Brazil; 4 Universidade Federal de Minas Gerais, Departamento de Medicina Preventiva e Social, Belo Horizonte, MG, Brasil Universidade Federal de Minas Gerais Universidade Federal de Minas Gerais Departamento de Medicina Preventiva e Social Belo Horizonte MG Brazil

**Keywords:** Minorias Sexuais e de Gênero, COVID-19, Comportamento Sedentário, Tabagismo, Consumo de Bebidas Alcoólicas, Estudos Transversais., Sexual and Gender Minorities, COVID-19, Sedentary Behavior, Tobacco Use Disorder, Alcohol Drinking, Cross-Sectional Studies., Minorías Sexuales y de Género, COVID-19, Conducta Sedentaria, Tabaquismo, Consumo de Bebidas Alcohólicas, Estudios Transversales.

## Abstract

**OBJETIVO:**

Verificar fatores associados à piora do estilo de vida, incluindo atividade física e consumo de cigarros e álcool, durante a pandemia de COVID-19, entre lésbicas, *gays*, bissexuais, transexuais, travestis e identidades relacionadas, Brasil, 2020.

**MÉTODOS:**

Estudo transversal, com indivíduos ≥18 anos de idade. *Odds ratio* (OR) e intervalos de confiança de 95% (IC_95%)_ foram estimados pela regressão logística.

**RESULTADOS:**

Dos 975 participantes, 48,9% (IC_95%_ 45,7;52,1) diminuíram sua atividade física; 6,2% (IC_95%_ 4,8;7,9) e 17,3% (IC_95%_ 15,0;19,8) aumentaram o consumo de cigarros e de álcool, respectivamente. Houve piora na realização de atividade física nos que aderiram às máscaras (OR=2,26; IC_95%_ 1,20;4,23), piora no consumo de cigarros naqueles com alguma condição crônica (OR=2,39; IC_95%_ 1,03;5,56) e de álcool nas mulheres cis (OR=1,95; IC_95%_ 1,31;2,92) e indivíduos morando com companheiro(a) (OR=1,89; IC_95%_ 1,23;2,91)

**CONCLUSÃO:**

Destacou-se piora do estilo de vida em mulheres cis, indivíduos com uma condição crônica e aqueles que aderiram às máscaras.

## Abstract (en)

**OBJECTIVE:**

To verify factors associated with deteriorating lifestyle during the COVID-19 pandemic, including physical activity, cigarette and alcohol intake in lesbians, gays, bisexuals, transsexuals, transvestites and people with related identities (LGBT+).

**METHODS:**

This was a cross-sectional study with individuals aged ≥18 years. Logistic regression was used to estimate odds ratios (OR) and 95% confidence intervals (95%CI).

**RESULTS:**

Of the 975 participants, 48.9% (95%CI 45.7;52.1) decreased physical activity, 6.2% (95%CI 4.8;7.9) increased cigarette smoking, and 17.3% (95%CI 15.0;19.8) increased alcohol intake. Physical activity deteriorated among individuals who adhered to mask use (OR=2.26; 95%CI 1.20;4.23), cigarette smoking increased among individuals who had a chronic health condition (OR=2.39; 95%CI 1.03;5.56), and alcohol intake increased among cisgender women (OR=1.95; 95%CI 1.31;2.92) and individuals living with a partner (OR=1.89; 95%CI 1.23;2.91).

**CONCLUSION:**

Lifestyle deterioration stood out among cisgender women, individuals with a chronic health condition and those who adhered to mask use.

## Resumen

**OBJETIVO:**

Verificar factores asociados al empeoramiento del estilo de vida durante la pandemia, incluida actividad física, consumo de cigarrillos y alcohol, en lesbianas, gays, bisexuales, transexuales, travestis e identidades relacionadas (LGBT+).

**MÉTODOS:**

Estudio transversal realizado en Brasil en agosto-noviembre, 2020, con individuos ≥18 años. Se utilizó regresión logística para estimar *odds ratio* (OR) y intervalos de confianza del IC_95%_.

**RESULTADOS:**

De 975 participantes, 48,9% (IC_95%_ 45,7;52,1) disminuyó la práctica de actividad física, 6,2% (IC_95%_ 4,8;7,9) aumentó el consumo de cigarrillos y 17,3% (IC_95%_ 15,0;19,8) aumentó el consumo de alcohol. Hubo empeoramiento en la actividad física entre individuos que adhirieron a mascarillas (OR=2,26; IC_95%_ 1,20;4,23), empeoramiento en consumo de cigarrillos entre individuos con una condición crónica (OR=2,39; IC_95%_ 1,03;5,56), y de alcohol entre mujeres-cis (OR=1,95; IC_95%_ 1,31;2,92) y personas que vivían en pareja (OR=1,89; IC_95%_ 1,23;2,91).

**CONCLUSIÓN:**

Se destacó empeoramiento en mujeres cis, individuos con una condición crónica y que adhirieron a las mascarillas.

## INTRODUÇÃO

**Table:** 

Contribuições do estudo	
Principais resultados	Verificou-se piora mais acentuada na atividade física do que no consumo de cigarros e de álcool em indivíduos LGBT+. Fatores associados a essas pioras foram aderir às máscaras, ter uma condição crônica, ser mulheres cis e morar com companheiro(a).
Implicações para os serviços	O prolongamento da pandemia pode impactar o ritmo de degradação da saúde física da população LGBT+. Perguntas sobre o ambiente social e a identidade de gênero durante as consultas de rotina podem ajudar a identificar indivíduos mais vulneráveis.
Perspectivas	O preenchimento dos dados sobre orientação sexual e identidade de gênero deve ser estimulado nos serviços de saúde, para que possa subsidiar políticas públicas voltadas para a população LGBT+ mais vulnerável durante a pandemia.

A rápida disseminação e elevada gravidade do coronavírus subtipo 2, ou SARS-CoV-2, levou a Organização Mundial da Saúde (OMS), em 11 de março de 2020, a declarar a pandemia da doença, denominada pela OMS como *Coronavirus Disease 2019* (COVID-19).^1^ No Brasil, a declaração da transmissão comunitária ocorreu em 20 de março de 2020,^2^ quando foram iniciadas as primeiras medidas de distanciamento social. Até o dia 24 de maio de 2021, o Brasil registrou 16.120.756 casos de COVID-19 e 449.858 óbitos pela doença.^3^

Em decorrência do distanciamento social, algumas vulnerabilidades, inerentes à população de lésbicas, *gays*, bissexuais, transexuais e identidades relacionadas (LGBT+), tornaram-se mais evidentes.^4^ Destaca-se a preocupação com a capacidade de esses segmentos sociais realizarem o distanciamento social, uma atitude dependente de fatores relacionados a tipo de contrato de trabalho, modelo de moradia e características socioculturais.^5^

O estilo de vida saudável é definido como um modo de viver que diminui o risco de contrair doenças e a morte precoce, principalmente quando se trata de doenças cardiovasculares e do câncer de pulmão, e inclui comportamentos, como a prática de atividade física regular, o consumo de cigarros e o consumo de bebidas alcoólicas.^6^ Dados de 45.161 brasileiros da população geral, com 18 ou mais anos de idade, coletados durante a pandemia, apontaram uma redução de 18% na prática de atividade física, associada ao maior consumo de alimentos ultraprocessados, como chocolates e biscoitos doces (5,8%), e de bebidas alcoólicas (17,6%). Na mesma amostra, considerados apenas os fumantes, 34% deles relataram aumento do número de cigarros fumados.^7^

Historicamente, a população LGBT+ (lésbicas, *gays*, bissexuais, transexuais, travestis e identidades relacionadas) apresenta maiores índices de consumo de cigarros e de bebida alcóolica.^8^^,^^9^ Este pior estilo de vida é parcialmente explicado pela teoria do estresse de minorias,^10^ e acarreta uma vulnerabilidade ainda maior à COVID-19. O pior estilo de vida está também relacionado a uma maior propensão do comprometimento da saúde mental de LGBT+s durante a pandemia, conforme demonstrado no Brasil^11^ e em outros países.^12^ Apesar disso, dados relativos ao estilo de vida dessa população ainda são escassos. Um estudo cuja população foi composta por homens que fazem sexo com homens e mulheres transgênero, todos imigrantes latinos nos Estados Unidos, evidenciaram um aumento de 23,1% no consumo de bebidas alcóolicas no período da pandemia da COVID-19.^13^ Semelhantemente,^14^ estudo realizado no Brasil identificou uma proporção de 29,7% de HSH e transgêneros que aumentaram a frequência do consumo de álcool durante a pandemia. Entretanto, esses estudos não exploraram os fatores predisponentes à piora do estilo de vida no que se refere a prática de atividade física regular, consumo de cigarro e consumo de álcool.

Segundo a OMS, a atividade física ajuda a melhorar a saúde mental^15^ e o controle do estresse durante o período de distanciamento social.^5^ Por sua vez, hábitos nocivos à saúde podem piorar a saúde mental.^5^ O tabagismo, além de ocasionar deterioração das condições do trato respiratório, aumenta a predisposição para COVID-19 grave.^7^ O consumo excessivo de álcool, por sua vez, provoca um enfraquecimento do sistema imunológico,^16^ e assim aumenta a vulnerabilidade ao SARS-CoV-2. Além disso, o álcool é um potente depressor do sistema nervoso central, exacerbando episódios depressivos e de ansiedade durante o distanciamento social.^17^

Ainda não há estudos no Brasil relativos à população LGBT+ no contexto da pandemia que abordem a piora do estilo de vida e seus fatores predisponentes. Essas informações poderiam auxiliar profissionais da Saúde na definição de grupos-alvo de ações de educação em saúde, entre a população LGBT+, e assim contribuir com políticas públicas para minimizar os impactos à saúde desses grupos, em tempos de pandemia da COVID-19.

Este estudo objetivou verificar fatores associados à piora do estilo de vida durante a pandemia de COVID-19 na população LGBT+, considerando-se a prática de atividade física regular, o consumo de cigarros e o consumo de álcool.

## MÉTODOS

Trata-se de estudo transversal realizado no Brasil, no período de 19 de agosto a 30 de novembro de 2020, com população-alvo constituída por indivíduos identificados como brasileiros pertencentes ao segmento LGBT+, com base em inquérito *online*.

O estudo pautou-se em dados do inquérito de saúde LGBT+, cujos objetivos foram caracterizar a população LGBT+ durante a pandemia da COVID-19 e especificar as características da pandemia nessa população. O inquérito de saúde LGBT+ é um estudo anônimo, realizado *online*, reunindo informações sociodemográficas, de saúde, sexualidade e discriminação social.^18^ Embora não representativo, o inquérito foi divulgado nacionalmente, em redes sociais como Facebook, Instagram e Whatsapp, utilizando-se um *link* de disseminação de tipo “bola de neve”,^19^ pelo qual um participante indica o estudo para outro, e assim por diante. Ainda, matérias sobre a pesquisa foram publicadas nos sítios eletrônicos oficiais das universidades participantes, incluídas a Universidade Federal de Minas Gerais (UFMG) e a Universidade Federal do Rio de Janeiro (UFRJ), além da imprensa de rádio, e *online*, para alcance de um maior número de pessoas.

Foi incluído no estudo todo indivíduo dessa população que preencheu os critérios de inclusão, quais sejam, ter 18 anos ou mais de idade, morar em uma das cinco grandes regiões brasileiras, autodeclarar-se pertencente à população LGBT+ e dispor de acesso à internet, de maneira a estar apto para preencher o questionário; e, naturalmente, ter concordado em participar da pesquisa.

Uma vez que o inquérito não foi desenhado especificamente para as variáveis dependentes deste estudo, o cálculo da amostra baseou-se na prevalência conservadora dos principais desfechos a serem estudados (50%; erro amostral de 5%), considerando-se o nível de significância de 99%. Assim definiu-se, como mínima, uma amostra de 664 respondentes.^18^

O mesmo inquérito foi dividido em quatro blocos: sociodemográfico; sexualidade; violência/discriminação; e condições de saúde. As variáveis consideradas no estudo foram extraídas dos blocos sociodemográfico e de condições de saúde. As variáveis dependentes foram aquelas relacionadas às características do estilo de vida durante a pandemia:

- prática de atividade física regular (não pratica; melhora ou manutenção; piora);
- consumo de cigarros (não fuma; melhora ou manutenção; piora); e
- consumo de álcool (não bebe; melhora ou manutenção; piora).

As variáveis independentes foram:

a) Características de gênero
- orientação afetiva (homossexual; bissexual; outras minorias de orientação afetiva);
- identidade de gênero (homem cis; mulher cis; transexual, travesti, não binário ou outras minorias de gênero).
b) Características sociodemográficas
- faixa etária (em anos: 18 a 29; 30 a 49; 50 ou mais);
- escolaridade (até a graduação; pós-graduação);
- raça/cor da pele (não branca; branca);
- morar com companheiro(a) (não; sim);
- número de moradores no domicílio (1; ≥2);
- região brasileira (Sudeste; outra); e
- receber auxílio emergencial do governo durante a pandemia (não; sim).
c) Características relacionadas à saúde
- número de condições crônicas (0; 1; ≥2);
- depressão (não; sim);
- pessoa próxima diagnosticada, atual ou previamente, com COVID-19 (não; sim).
- adesão ao distanciamento social (não; sim); e
- adesão ao uso de máscara facial (não; sim).

Todas essas características foram mensuradas pela percepção do indivíduo e comparadas às mesmas características referidas para o momento prévio à COVID-19, em relação à piora, melhora ou manutenção de seu estilo de vida no período da pandemia.

O respondente foi indagado quanto (i) à prática de atividades físicas regularmente, e o tempo gasto nessas atividades em minutos, (ii) ao consumo e quantidade de cigarros fumados por dia, e (iii) ao consumo de álcool antes do período da pandemia, e a frequência desse comportamento em dias/semana, além da quantidade de doses de bebida alcóolica ingeridas por ocasião. Diante dessas respostas, para os que responderam praticar atividade física regular, por exemplo, propôs-se a seguinte pergunta: *Após o início da pandemia de COVID-19, você conseguiu manter a frequência e as horas de atividade física descritas anteriormente?*, cujas opções de resposta foram ‘sim, mantive igual’, ‘não, diminuí a frequência e/ou horas de atividade’ e ‘não, aumentei a frequência e/ou horas de atividade’. Questões similares foram apresentadas relativamente ao consumo de cigarros e ao consumo de álcool, para aqueles que relataram esses consumos antes da pandemia. Voltando à prática de atividade física regular, aqueles que responderam ter diminuído a frequência e/ou horas dessa atividade foram classificados na categoria de ‘piora’, enquanto os que responderam ter aumentado ou mantido a frequência e/ou horas de atividade física foram classificados na categoria de ‘melhora ou manutenção’ desse comportamento. Para o consumo de cigarros e o de álcool, os que referiram ter aumentado o número de cigarros e a frequência e/ou doses de álcool consumidos foram classificados em ‘piora’, enquanto aqueles que responderam ter diminuído ou mantido o número de cigarros e a frequência e/ou doses de álcool consumida, considerados em ‘melhora ou manutenção’.

As categorias das regiões brasileiras foram escolhidas considerando-se a composição amostral predominante da região Sudeste.^18^ As condições crônicas foram as diagnosticadas por um médico, entre as quais foram incluídas diabetes *mellitus*, hipertensão, doença do coração, acidente vascular encefálico, doença respiratória, doença autoimune, insuficiência renal crônica e câncer. A depressão foi considerada separadamente das demais condições crônicas, pois a saúde mental é uma questão importante para a população LGBT+.^11^ Finalmente, sobre a adesão ao distanciamento social e ao uso de máscaras, o respondente tinha de concordar totalmente com ter seguido as recomendações das instituições sanitárias responsáveis.

Primeiramente, realizou-se uma análise descritiva dos dados da amostra, por meio de frequências absolutas e relativas. Diferenças heterogêneas entre as categorias de piora do estilo de vida durante a pandemia da COVID-19 foram realizadas pelo teste qui-quadrado de Pearson. A regressão logística foi utilizada para estimar as *odds ratio* (OR) ou razões de chances, e intervalos de confiança de 95% (IC_95%_), no sentido de investigar os fatores associados à piora do estilo de vida entre a população LGBT+, ao longo da pandemia. Nessa análise, foram incluídos tão somente os indivíduos que, antes da emergência da COVID-19, já apresentavam os estilos de vida selecionados, quais sejam, prática de atividade física, consumo de cigarros e consumo de álcool, adotando-se como categoria de referência a manutenção ou melhora do respectivo estilo de vida. Para cada um dos desfechos, foram criados modelos estatísticos separados. As variáveis com p<0,20 nos modelos brutos foram incluídas nos modelos ajustados. O teste de Hosmer-Lemeshow foi aplicado para analisar a adequação dos modelos finais, e o teste de multicolinearidade, para testar a correlação entre as variáveis incluídas nos modelos finais. Todas as análises estatísticas utilizaram-se do *software* Stata 14.2 SE (Stata-Corp., College Station, Texas, USA).

O inquérito de saúde LGBT+ foi aprovado em 6 de agosto de 2020, pelo Comitê de Ética em Pesquisa da Universidade Federal de Minas Gerais (CEP/UFMG): Certificado de Apresentação para Apreciação Ética (CAAE) n^o^ 34123920.9.0000.5149; Parecer n^o^ 4.198.297. Por ser um questionário anônimo, solicitou-se a dispensa do Termo de Consentimento Livre e Esclarecido. Entretanto, os pesquisadores realizaram o processo de consentimento, que consistiu de uma breve descrição dos objetivos e desenho da pesquisa, forneceram o contato de um dos pesquisadores responsáveis e informaram sobre potenciais riscos e benefícios para os consultados.

## RESULTADOS

**Figura 1:**
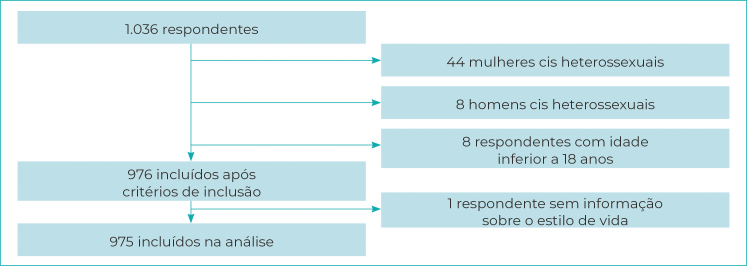
- Composição final da amostra do estudo após a aplicação dos critérios de inclusão, Brasil, agosto-novembro de 2020

Dos 1.036 indivíduos LGBT+ que concordaram em responder ao inquérito *online*, 976 atendiam aos critérios de inclusão, e destes, 975 referiram ter informação a respeito das variáveis dependentes (prática de atividade física regular, consumo de cigarros e consumo de álcool), sendo incluídos na presente análise (Figura 1).

Para seis variáveis independentes, faltavam alguns dados, a variar entre 1 e 13, uma vez que a resposta a cada uma das perguntas dos blocos do inquérito não era obrigatória. Em relação à piora do estilo de vida durante a pandemia, 48,9% (IC_95%_ 45,7;52,1) diminuíram a prática de atividade física regular, 6,2% (IC_95%_ 4,8;7,9) aumentaram o consumo de cigarros e 17,3% (IC_95%_ 15,0;19,8) aumentaram o consumo de álcool. A média de idade dos participantes foi de 31,3 anos (desvio-padrão: 11,5).

A Tabela 1 descreve as características de todos os respondentes segundo a piora do estilo de vida durante a pandemia. A maioria correspondia a homossexuais (72,2%), era de raça/cor da pele branca (60,1%), e exatamente a metade era de homens cis (50,0%). A faixa etária mais frequente foi a dos 18 aos 29 anos (55,4%), e a escolaridade predominante, até a graduação (68,4%). Maior frequência de piora na prática de atividade física regular foi encontrada em mulheres cis, indivíduos nas faixas etárias de 30 a 49 anos e de 50 anos ou mais, na escolaridade mais alta (pós-graduação), naqueles que não recebiam auxílio emergencial do governo durante a pandemia, entre os que não tinham depressão, e para os que não referiram pessoa próxima diagnosticada - atual ou previamente - com COVID-19.

**Tabela 1:** - Características de todos os respondentes do inquérito Saúde LGBT+ (n=975) segundo a piora do estilo de vida durante a pandemia, Brasil, agosto-novembro de 2020

Variáveis		Piora do estilo de vida durante a pandemia			
	Total	Prática de atividade física regular	Consumo de cigarros	Consumo de álcool
	n (%)	n (%)	n (%)	n (%)
Características de gênero				
Orientação afetiva				
Homossexual	704 (72,2)	341 (48,5)	47 (6,7)	128 (18,3)
Bissexual	232 (23,8)	117 (50.4)	13 (5,7)	36 (15,6)
Outras minorias de orientação afetiva	39 (4,0)	18 (47,4)	-	4 (10,3)
Identidade de gênero				
Homem cis	487 (50,0)	178 (44,1)	33 (8,2)^a^	82 (20,3)
Mulher cis	405 (41,5)	259 (53,3)^a^	20 (4,1)	72 (14,9)
Transexual, travesti, não binário ou outras minorias de gênero	83 (8,5)	39 (47,0)	7 (8,5)^a^	14 (16,9)
Características sociodemográficas				
Faixa etária (anos)				
18-29	540 (55,4)	240 (44,5)	29 (5,4)	91 (16,9)^a^
30-49	336 (34,5)	183 (54,6)^a^	25 (7,4)	66 (19,8)^a^
≥50	98 (10,1)	53 (54,1)^a^	6 (6,2)	11 (11,2)
Escolaridade				
Até a graduação	667 (68,4)	303 (45,5)	43 (6,5)	106 (16,0)
Pós-graduação	308 (31,6)	173 (56,4)^a^	17 (5,5)	62 (20,2)
Raça/cor da pele^b^				
Não branca	389 (39,9)	192 (49,5)	22 (5,7)	62 (16,0)
Branca	585 (60,1)	283 (48,5)	38 (6,5)	106 (18,2)
Morar com companheiro(a)^c^				
Não	718 (74,2)	343 (47,9)	41 (5,8)	113 (15,8)
Sim	250 (25,8)	128 (51,2)	19 (7,6)	53 (21,3)^a^
Número de moradores no domicílio^d^				
1	195 (20,2)	98 (50,3)	16 (8,2)	42 (21,8)
≥2	772 (79,8)	373 (48,4)	43 (5,6)	126 (16,4)
Região brasileira^b^				
Sudeste	781 (80,2)	384 (49,2)	49 (6,3)	131 (16,8)
Outra	193 (19,8)	91 (47,6)	11 (5,8)	37 (19,3)
Receber auxílio emergencial do governo durante a pandemia^e^				
Não	703 (72,8)	360 (51,4)^a^	45 (6,4)	133 (19,0)^a^
Sim	262 (27,2)	111 (42,4)	15 (5,8)	35 (13,4)
Características relacionadas à saúde				
Número de condições crônicas^f^				
0	504 (52,4)	256 (51,0)	20 (4,0)	80 (15,9)
1	344 (35,8)	166 (48,3)	31 (9,1)^a^	68 (19,9)
≥2	114 (11,8)	46 (40,4)	9 (7,9)	19 (16,7)
Depressão^f^				
Não	725 (75,4)	363 (50,2)^a^	35 (4,8)	119 (16,5)
Sim	237 (24,6)	105 (44,3)	25 (10,6)^a^	48 (20,3)^a^
Pessoa próxima diagnosticada atual ou previamente com COVID-19^b^				
Sim	227 (23,3)	94 (41,4)	17 (7,6)	36 (15,9)
Não	747 (76,7)	382 (51,3)^a^	43 (5,8)	132 (17,7)
Adesão ao distanciamento social				
Não	376 (38,6)	184 (48,9)	31 (8,3)^a^	89 (23,8)^a^
Sim	599 (61,4)	292 (48,9)	29 (4,9)	79 (13,2)
Adesão ao uso de máscara facial				
Não	142 (14,6)	61 (43,0)	8 (5,7)	35 (25,0)^a^
Sim	833 (85,4)	415 (49,9)	52 (6,3)^a^	133 (16,0)
N Total	975	476	60	168

a) Categorias com frequência de piora do estilo de vida estatisticamente maior (p<0,05), de acordo com o teste qui-quadrado de Pearson; dados faltantes por ausência de resposta: b) 1; c) 7; d) 12; e) 10; f) 13.

Maior frequência de piora do consumo de cigarros foi encontrada em homens cis e transexuais, travestis, não binários ou outras minorias de gênero, indivíduos que apresentavam uma única condição crônica e os que tinham depressão. Ainda, aqueles que não aderiram ao distanciamento social e os que aderiram ao uso de máscaras também apresentaram maior frequência de piora no consumo de cigarros. Por último, maior frequência de piora do consumo de álcool foi encontrada entre os mais jovens (faixas etárias até 49 anos), que moravam com companheiro(a), que não recebiam auxílio emergencial do governo durante a pandemia, que tinham depressão, que não aderiram ao distanciamento social, assim como os que não aderiram ao uso de máscaras.

A Tabela 2 mostra os resultados dos modelos brutos e ajustados dos fatores associados a cada uma das características de piora do estilo de vida durante a pandemia, considerando-se somente os indivíduos que já apresentavam aquele estilo de vida antes da emergência da COVID-19. Como a multicolinearidade não foi evidenciada em nenhum dos modelos ajustados (fator de inflação de variância <2), todas as variáveis independentes com p<0,20 nos modelos brutos foram mantidas nos modelos ajustados. Todos os modelos apresentaram p>0,05 no teste de Hosmer-Lemeshow, revelando adequação. Relativamente à piora da prática de atividade física regular, frequência estatisticamente maior foi observada apenas entre aqueles que aderiram ao uso de máscara (OR=2,26; IC_95%_ 1,20;4,23). Entre os que apresentaram piora no consumo de cigarros, frequência estatisticamente maior foi observada tão somente nos indivíduos com uma condição crônica (OR=2,39; IC_95%_ 1,03;5,56), frente àqueles com nenhuma condição crônica. Quanto à piora do consumo de álcool, frequência estatisticamente maior foi observada nas mulheres cis (OR=1,95; IC_95%_ 1,31;2,92), em relação aos homens cis, e para a variável ‘morar com companheiro(a)’ (OR=1,89; IC_95%_ 1,23;2,91), em relação a não morar com companheiro(a). Em contrapartida, frequências estatisticamente menores de piora do consumo de álcool foram observadas na faixa etária ≥50 anos (OR=0,42; IC_95%_ 0,19;0,89), em relação à faixa de 18-29 anos, naqueles que recebiam auxílio emergencial do governo durante a pandemia (OR=0,52; IC_95%_ 0,33;0,83) e nos que aderiam ao distanciamento social (OR=0,51; IC_95%_ 0,35;0,75).

**Tabela 2:** - Modelos brutos e ajustados da associação entre as características dos respondentes do inquérito de saúde LGBT+ e a piora do estilo de vida durante a pandemia de COVID-19, Brasil, agosto-novembro de 2020

Características	Estilo de vida durante a pandemia							
	Diminuição da prática de atividade física regular (*versus* manteve ou aumentou) N=533			Aumento do consumo de cigarros (*versus* manteve ou diminuiu) N=198			Aumento do consumo de álcool (*versus* manteve ou diminuiu) N=778	
	OR^a^ brutas (IC_95%_^b^)	OR ajustadas^c^ (IC_95%_)		OR brutas (IC_95%_)	OR ajustadas^d^ (IC_95%_)		OR brutas (IC_95%_)	OR ajustadas^e^ (IC_95%_)
Características de gênero								
Orientação afetiva (*versus*^f^homossexual)								
Bissexual	0,91 (0,53;1,58)	-		0,78 (0,38;1,61)	-		0,81 (0,54;1,22)	-
Outras minorias de orientação afetiva	2,96 (0,37;22,58)	-		-	-		0,96 (0,31;2,94)	-
**Identidade de gênero (*versus* homem cis)**								
Mulher cis	0,69 (0,41;1,14)	0,64 (0,37;1,10)		1,45 (0,75;2,80)	-		1,46 (1,03;2,09)^f^	1,95 (1,31;2,92)^f^
Transexual, travesti, não binário ou outras minorias de gênero	0,66 (0,28;1,52)	0,54 (0,22;1,12)		1,29 (0,46;3,59)	-		1,51 (0,78;2,90)	1,56 (0,78;3,24)
Características sociodemográficas								
**Faixa etária (*versus* 18-29 anos)**								
30-49 anos	1,23 (0,73;2,08)	1,63 (0,85;3,11)		1,41 (0,73;2,69)	-		1,32 (0,92;1,89)	1,04 (0,66;1,63)
≥50 anos	0,93 (0,44;1,97)	1,12 (0,48;2,61)		0,74 (0,27;2,01)	-		0,70 (0,35;1,37)	0,42 (0,19;0,89)^f^
Pós-graduação (*versus* até a graduação)	0,87 (0,53;1,41)	0,70 (0,38;1,28)		1,08 (0,55;2,12)	-		1,40 (0,98;2,00)	1,06 (0,68;1,65)
Raça/cor da pele branca (*versus* não branca)	0,66 (0,39;1,10)	-		1,19 (0,64;2,22)	-		1,12 (0,79;1,59)	-
Morar com companheiro(a) (*versus* não)	1,71 (0,92;3,15)	-		1,70 (0,86;3,35)	1,90 (0,91;3,93)		1,59 (1,09;2,31)^f^	1,89 (1,23;2,91)^f^
Número de moradores no domicílio ≥2 (*versus* 1)	1,49 (0,87;2,57)	1,61 (0,89;2,91)		0,69 (0,34;1,39)	-		0,72 (0,48;1,07)	0,65 (0,41;1,04)
Região Sudeste (*versus* outra)	0,84 (0,45;1,60)	-		0,85 (0,37;1,83)	-		0,82 (0,54;1,24)	0,85 (0,54;1,32)
Receber auxílio emergencial do governo durante a pandemia (*versus* não)	1,52 (0,81;2,86)	1,38 (0,70;2,71)		0,59 (0,30;1,16)	0,55 (0,27;1,13)		0,63 (0,42;0,95)^f^	0,52 (0,33;0,83)^f^
Características relacionadas à saúde								
**Número de condições crônicas (*versus* nenhuma)**								
1	1,33 (0,76;2,26)	1,45 (0,84;2,52)		2,61 (1,33;5,11)^f^	2,39 (1,03;5,56)^f^		1,36 (0,94;1,96)	1,32 (0,84;2,09)
≥2	1,69 (0,64;4,47)	1,69 (0,62;4,59)		1,39 (0,56;3,45)	0,93 (0,28;3,05)		1,33 (0,75;2,35)	1,35 (0,64;2,85)
Depressão (*versus* não)	1,52 (0,79;2,92)	-		1,89 (1,01;3,55)^f^	1,27 (0,53;3,07)		1,43 (0,97;2,10)	1,14 (0,67;1,95)
Pessoa próxima diagnosticada atual ou previamente com COVID-19 (*versus* não)	1,31 (0,74;2,30)	-		0,63 (0,31;1,26)	0,60 (0,29;1,27)		1,10 (0,73;1,66)	-
Adesão ao distanciamento social (*versus* não)	0,71 (0,42;1,18)	-		0,70 (0,38;1,29)	-		0,50 (0,36;0,71)^f^	0,51 (0,35;0,75)^f^
Adesão ao uso de máscara facial (*versus* não)	1,90 (1,04;3,46)^f^	2,26 (1,20;4,23)^f^		1,82 (0,78;4,22)	2,08 (0,85;5,12)		0,55 (0,35;0,85)^f^	0,64 (0,39;1,06)

a) OR: *odds ratio* (razão de chances); b) IC_95%_: intervalo de confiança de 95%; Adequação dos modelos: c) 130,9 - p=0,41; d) 51,8 - p=0,08; e) 387,0 - p=0,15; f) valores de p<0,05 com base na regressão logística.

## DISCUSSÃO

Os resultados desta pesquisa sugerem que alguns grupos de indivíduos LGBT+ tiveram piora do estilo de vida quando submetidos ao ambiente imposto pela pandemia. Piora na prática de atividade física regular foi observada somente naqueles que aderiram ao uso de máscara. Piora no consumo de cigarros, por sua vez, foi observada apenas em quem referiu uma única condição crônica. Relativamente à piora do consumo de álcool, resultados mostraram frequências maiores entre as mulheres cis e os(as) que moravam com companheiro(a). Ainda sobre a piora do consumo de álcool, essa constatação mostrou-se negativamente associada à faixa etária dos 50 anos ou mais, ao recebimento de auxílio emergencial do governo durante a pandemia e à adesão ao distanciamento social.

Os resultados mostraram que, entre os praticantes de atividade física regular, a piora desse comportamento foi maior entre aqueles que aderiram ao uso de máscara. Um estudo brasileiro mostrou que adultos mais velhos e que mantinham hábitos de vida saudáveis antes da pandemia tenderam a aderir mais às medidas preventivas relacionadas à COVID-19.^20^ Entretanto, aqueles que praticavam atividade física aderiram menos ao uso de máscaras, sugerindo que essa medida pode dificultar a manutenção da prática dessas atividades, devido ao desconforto para respirar.^20^ Cabe ressaltar que, neste estudo, não foram avaliadas mudanças nos estilos de vida.

O momento atual de pandemia, pode, por si só, contribuir para a redução do número de praticantes e horas dedicadas à atividade física regular. Os resultados encontrados revelam quase 50% da população LGBT+ tendo reduzido a prática de atividade física regular, enquanto, na população geral brasileira da mesma faixa etária, essa redução foi de 18%.^7^ Em 2020, Malta et al. compararam o período da pandemia aos feriados e férias, comumente datas de relaxamento dos indivíduos, resultando em uma redução de movimentação e aumento de horas gastas com programas televisivos e navegação na internet, o que caracteriza um maior comportamento sedentário nestes períodos.^7^ Apesar disso, segundo estudo de Guimarães Lima et al., realizado em 2019,^21^ a caminhada, o futebol e a musculação, justamente as modalidades mais praticadas no Brasil, vão de encontro às medidas de distanciamento social, já que são praticadas, principalmente, em espaços públicos, o que promove o contato entre seus praticantes. Segundo a teoria da escala denominada Motives for Physical Activity Measure - Revised (MPAM-R),^22^ que pode de ser utilizada para medir a motivação dos indivíduos para a realização de atividade física, a convivência com grupos é determinante nesse sentido. Considerando-se o cenário pandêmico, essas atividades coletivas ficaram bastante comprometidas, o que poderia impactar na decisão dos indivíduos de reduzir a prática de atividade física.

Em relação ao aumento do consumo de álcool, o comprometimento da saúde mental pode explicar tal comportamento,^23^^-^^25^ seja pelo medo de contaminação, seja pela necessidade de distanciamento social ou, ainda, pela redução da renda.^26^ Os resultados do estudo em tela apontaram aumento de 17,3% no consumo de bebidas alcoólicas durante a pandemia, semelhante ao da população geral brasileira (17,6%),^7^ embora menor que o reportado nos meses de abril e maio de 2020 (prévios à instalação da pandemia no território nacional), especialmente entre seu contingente LGBT+.^14^ Quanto ao consumo de tabaco, encontrou-se um aumento de 42% entre os fumantes (dados não apresentados), semelhante a outros dados proporcionais da população brasileira LGBT+ (49,4%)^14^ e maior que os da população brasileira geral (34%).^7^ Estudo realizado na população geral da Austrália, em abril de 2020, encontrou, durante a pandemia, associação entre piora do estilo de vida e depressão, ansiedade e sintomas de estresse, condições que, na maior parte das vezes, são mais proeminentes entre as mulheres^25^ e podem explicar o aumento do consumo de álcool.^25^ Diferentemente do que foi reportado antes da pandemia, no que concerne a homens que fazem sexo com homens e transgêneros brasileiros,^14^ os achados do presente estudo evidenciaram maior consumo de álcool apenas entre as mulheres. Para mulheres com identidades de gênero minoritárias (incluindo mulheres cisgênero, lésbicas ou bissexuais, e não binárias), o conteúdo postado em redes sociais e relacionado ao consumo de álcool passou de um padrão ‘associado a festas’ para um padrão de ‘socialização e superação da pandemia’.^27^ Estes achados reforçam o uso do álcool como um importante mecanismo compensatório para lidar com a pandemia, nesse grupo.

Os grupos de idade mais avançada (≥50 anos), bem como os que recebiam auxílio emergencial do governo durante a pandemia e que aderiram ao distanciamento social, apresentaram menor susceptibilidade ao aumento do consumo de álcool no período estudado, possivelmente uma consequência da oportunidade de manutenção da interação entre indivíduos. Brasileiros LGBT+ que consumiam cinco ou mais doses de álcool em uma única ocasião (*binge drinking*) mostraram maior dificuldade em aderir ao distanciamento social,^14^ segundo estudo cujo resultado corrobora o desta pesquisa. Além disso, maior sofrimento decorrente do distanciamento social foi reportado para os mais jovens,^25^^,^^28^ pertencentes ao chamado grupo potencialmente produtivo (entre os 15 e os 59 anos de idade).^28^ Fora do período da pandemia, espera-se que esses indivíduos tenham maior interação social e, portanto, em um momento de distanciamento obrigatório, sejam os mais impactados mentalmente, pela COVID-19.^25^^,^^28^ Entre os mais vulneráveis, especialmente aqueles que recebiam auxílio emergencial do governo, apesar de também serem consideravelmente impactados em sua saúde mental, pelo desemprego e dificuldade financeira,^5^ isso não se refletiu em piora do consumo de álcool. Grupos mais vulneráveis tiveram de priorizar sua alimentação^5^ e a manutenção da moradia, restando menos recursos financeiros disponíveis para o consumo de álcool.

Apesar de apresentar pontos fortes, como ser a primeira pesquisa de âmbito nacional a investigar o tema e destacar a piora do estilo de vida em uma população historicamente negligenciada, algumas limitações devem ser levantadas. Primeiramente, as limitações inerentes ao método de inquérito *online*, como a não representatividade e o efeito voluntário,^29^ excluindo-se a população mais vulnerável e sem acesso à internet. Desse modo, não se descarta viés de seleção e, por conseguinte, os resultados do estudo não se podem generalizar para toda a população LGBT+. Em segundo lugar, cabe o fato de não se ter avaliado a taxa de resposta, uma vez que o *software* utilizado não o permitia, ademais da duplicidade de informações, por se tratar de uma pesquisa anônima. Em terceiro lugar, não foram utilizados pesos para possíveis correções estatísticas visando ampliar a representatividade da população-alvo, dado que houve uma grande concentração de respondentes na região Sudeste. Em quarto lugar, o caráter transversal da pesquisa não permitiu o conhecimento da direção das associações encontradas. E finalmente, todos os desfechos foram mensurados a partir de autorrelato, passível de ser influenciado por outros fatores mentais não incluídos na pesquisa, além de estar sujeito à percepção individual e ao viés de informação.

Em conclusão, os resultados reforçam que os indivíduos identificados como pertencentes ao grupo LGBT+ apresentaram vulnerabilidade, quanto à piora do estilo de vida durante a pandemia da COVID-19. Entre os fatores associados a essa piora do estilo de vida, destacaram-se (i) os indivíduos que aderiram ao uso de máscaras, (ii) aqueles com uma condição crônica e (iii) as mulher cis. Estes resultados sugerem que o prolongamento da pandemia e das medidas de distanciamento social podem impactar o ritmo de degradação da saúde entre a população LGBT+.
