# Supplementary material for: Factors associated with lifestyle deterioration during the COVID-19 pandemic among Brazilian lesbians, gays, bisexuals, transsexuals, transvestites and related identities: a cross-sectional study
Source: Epidemiol Serv Saude. 2022 Feb 25;31(1):e2021752. [Article in Portuguese] doi: 10.1590/S1679-49742022000100005 (PMC11321200; doi:10.1590/S1679-49742022000100005)
Supplement: Translation

# Translation: Factors associated with lifestyle deterioration during the COVID-19 pandemic among Brazilian lesbians, gays, bisexuals, transsexuals, transvestites and related identities: a cross-sectional study

**Authors:** Luciana Helena Reis Braga, Cynthia Santos Menezes, Isadora Viegas Martins, Janderson Diego Pimenta da Silva, Juliana Lustosa Torres

**Affiliations:** 1 Universidade Federal de Minas Gerais, Departamento de Gestão de Serviços de Saúde, Belo Horizonte, MG, Brazil; 2 Universidade Federal de Minas Gerais, Departamento de Direito, Belo Horizonte, MG, Brazil; 3 Universidade do Estado de Mato Grosso, Departamento de Enfermagem, Tangará da Serra, MT, Brazil; 4 Universidade Federal de Minas Gerais, Departamento de Medicina Preventiva e Social, Belo Horizonte, MG, Brazil

**Keywords:** Sexual and Gender Minorities, COVID-19, Sedentary Behavior, Tobacco Use Disorder, Alcohol Drinking, Cross-Sectional Studies.

## Abstract

**OBJECTIVE:**

To verify factors associated with deteriorating lifestyle during the COVID-19 pandemic, including physical activity, cigarette and alcohol intake in lesbians, gays, bisexuals, transsexuals, transvestites and people with related identities (LGBT+).

**METHODS:**

This was a cross-sectional study with individuals aged ≥18 years. Logistic regression was used to estimate odds ratios (OR) and 95% confidence intervals (95%CI).

**RESULTS:**

Of the 975 participants, 48.9% (95%CI 45.7;52.1) decreased physical activity, 6.2% (95%CI 4.8;7.9) increased cigarette smoking, and 17.3% (95%CI 15.0;19.8) increased alcohol intake. Physical activity deteriorated among individuals who adhered to mask use (OR=2.26; 95%CI 1.20;4.23), cigarette smoking increased among individuals who had a chronic health condition (OR=2.39; 95%CI 1.03;5.56), and alcohol intake increased among cisgender women (OR=1.95; 95%CI 1.31;2.92) and individuals living with a partner (OR=1.89; 95%CI 1.23;2.91).

**CONCLUSION:**

Lifestyle deterioration stood out among cisgender women, individuals with a chronic health condition and those who adhered to mask use.

## INTRODUCTION

**Table:** 

Study contributions	
Main results	Physical activity decreased more than cigarette and alcohol intake increased among LGBT+ individuals. Factors association with these forms of lifestyle deterioration were adherence to facemask use, having a chronic health condition, being cisgender women and living with a partner.
Implications for services	The prolongation of the pandemic may impact the rate at which the physical health of LGBT+ people deteriorates. Questions about social environment and gender identity during routine medical consultations can help identify more vulnerable individuals.
Perspectives	The filling in of data on sexual orientation and gender identity should be encouraged in health services, so that such information can guide public policies aimed at more vulnerable LGBT+ people during the pandemic.

The rapid spread and high severity of coronavirus subtype 2, or SARS-CoV-2, led the World Health Organization (WHO) to declare, on March 11^th^ 2020, that the disease had gained pandemic proportions, naming it Coronavirus Disease 2019 (COVID-19). ^1^ In Brazil, community transmission of the disease was officially announced on March 20^th^ 2020,^2^ when the first social distancing measures began. By May 24^th^ 2021, Brazil had recorded 16,120,756 COVID-19 cases and 449,858 COVID-19 deaths. ^3^

As a result of social distancing, some vulnerabilities, inherent to the lesbian, gay, bisexual, transsexual and related identities (LGBT+) population, have become more evident. ^4^ There is noteworthy concern with the ability of these social segments to achieve social distancing, this being an attitude that depends on factors related to type of job, housing conditions and sociocultural characteristics. ^5^

Healthy lifestyle is defined as a way of living that decreases the risk of disease and early death, especially when it comes to cardiovascular disease and lung cancer, and includes behaviors such as regular physical activity, smoking and drinking. ^6^ Data collected during the pandemic on 45,161 general population Brazilians, aged 18 years or more, showed an 18% reduction in physical activity, associated with higher intake of ultra-processed food, such as chocolates and sweet cookies (5.8%) and alcoholic beverages (17.6%). In the same sample, considering only smokers, 34% of them reported an increase in the number of cigarettes they smoked. ^7^

Historically, higher rates of smoking and drinking are found among the LGBT+ population (lesbian, gay, bisexual, transsexual, transvestite and related identities). ^8^^,^^9^ This poorer lifestyle is partly explained by the minority stress theory, ^10^ and entails even greater vulnerability to COVID-19. Poorer lifestyle is also related to a greater tendency of LGBT+ mental health impairment during the pandemic, as demonstrated in Brazil^11^ and in other countries.^12^ Despite this, data regarding the lifestyle of this population is still scarce. A study conducted with men who have sex with men and transgender women, all Latino immigrants in the United States, showed a 23.1% increase in alcohol consumption during the COVID-19 pandemic period. ^13^ Similarly, a study conducted in Brazil with MSM and transgender persons^14^ identified that 29.7% of them increased their frequency of alcohol intake during the pandemic. However, these studies did not explore predisposing factors for lifestyle deterioration regarding regular physical activity, cigarette smoking and alcohol consumption.

According to the WHO, physical activity helps improve mental health^15^ and stress management while social distancing measures are in force. ^5^ In turn, unhealthy habits can worsen mental health.^5^ In addition to causing deterioration of respiratory tract conditions, smoking increases predisposition to contracting severe COVID-19.^7^ In turn, excessive alcohol intake weakens the immune system^16^ and as such increases vulnerability to SARS-CoV-2. In addition, alcohol is a potent central nervous system depressant, thus exacerbating episodes of depression and anxiety during social distancing.^17^

As yet there are no studies in Brazil concerning the LGBT+ population in the context of the pandemic that address lifestyle deterioration and its predisposing factors. This information could help health professionals in defining target groups for health education actions among the LGBT+ population, and thus contribute to public policies to minimize impact on the health of these groups during the COVID-19 pandemic.

This study aimed to verify factors associated with lifestyle deterioration among the LGBT+ population during the COVID-19 pandemic, considering regular physical activity, cigarette smoking and alcohol consumption.

## METHODS

This was a cross-sectional study conducted in Brazil, from August 19^th^ to November 30^th^ 2020, with a target population consisting of individuals identified as Brazilians who belong to the LGBT+ segment of the population, based on an online survey.

The study was based on data from the LGBT+ Health Survey, the objectives of which were to characterize the LGBT+ population during the COVID-19 pandemic and to specify the characteristics of the pandemic in this population. The LGBT+ Health Survey was an anonymous online survey that gathered sociodemographic, health, sexuality and social discrimination information.^18^ Although not representative, the survey was publicized nationally on social networks such as Facebook, Instagram and Whatsapp, using a “snowball” method to pass on the survey link^19^ whereby one participant indicates the survey to someone else and so on and so forth. In addition, news items about the survey were published on the official websites of the participating universities, including the Universidade Federal de Minas Gerais (UFMG) and the Universidade Federal do Rio de Janeiro (UFRJ), in addition to radio coverage and online stories, in order to reach the largest possible number of people.

The study included all people from this population who met the inclusion criteria, which were: being 18 years of age or older, living in one of Brazil’s five regions, self-reporting belonging to the LGBT+ population and having access to the internet in order to be able to fill out the questionnaire; and, of course, having agreed to take part in the study.

Given that the survey was not specifically designed for the dependent variables of this study, the sample size calculation was based on a conservative estimate of the prevalence of the main outcomes to be studied (50%; 5% sampling error), considering a 99% significance level. Thus the minimum sample size was defined as being 664 respondents.^18^

The survey itself was divided into four blocks of information: sociodemographic; sexuality; violence/discrimination; and health conditions. The variables considered in the study were retrieved from the sociodemographic and health conditions blocks of information. The dependent variables were those related to lifestyle characteristics during the pandemic:

- regular physical activity (does not do physical activity; increased or continues the same; deteriorated);
- cigarette smoking (does not smoke; increased or continues the same; got worse); and
- alcohol intake (does not drink; increased or continues the same; got worse).

The independent variables were:

a) Gender characteristics
- affective orientation (homosexual; bisexual; other affective orientation minorities);
- gender identity (cisgender male; cisgender female; transsexual, transvestite, non-binary or other gender minorities).
b) Sociodemographic characteristics
- age group (in years: 18-29; 30-49; 50 or over);
- schooling (up to degree level; postgraduate);
- race/skin color (non-white; white);
- living with a partner (no; yes);
- number of dwellers in household (1; ≥2);
- region of Brazil (Southeast; other); and
- receiving government emergency aid during the pandemic (no; yes).
c) Health-related characteristics
- number of chronic conditions (0; 1; ≥2);
- depression (no; yes);
- someone close currently or previously diagnosed as having COVID-19 (no; yes).
- adherence to social distancing (no; yes); and
- adherence to facemask use (no; yes).

All these characteristics were measured according to the individual’s perception of them and were compared with the same characteristics reported for the time prior to COVID-19, in terms of their lifestyle having deteriorated, improved or remained the same in the pandemic period.

Respondents were asked (i) whether or not they practiced physical activities regularly, and how long in minutes they spent doing them, (ii) whether or not they smoked and how many cigarettes they smoked a day, and (iii) whether or not they drank alcohol before the pandemic, and frequency of drinking expressed in days per week, as well as the number of units of alcoholic beverage drunk each time. Depending on their answers, those who answered that they did regular physical activity, for example, were asked the following: *After the COVID-19 pandemic started, were you able to maintain the frequency and time spent doing physical activity reported for the period prior to the pandemic?*, with the following answer options ‘yes, I continued the same’, ‘no, I decreased the frequency and/or time spent doing physical activity’, and ‘no, I increased the frequency and/or time spent doing physical activity’. Similar questions were asked about cigarette smoking and alcohol intake, in the case of those who reported smoking and/or drinking before the pandemic.

Returning to regular physical activity, those who reported having decreased frequency and/or time spent doing physical activity were classified as ‘deteriorating’, while those who reported having increased or maintained frequency and/or hours of physical activity were classified as ‘improved or continues the same’. In the case of cigarette and alcohol consumption, those who reported increasing the number of cigarettes and frequency and/or units of alcohol consumed were classified as ‘deteriorating’, while those who reported decreasing or maintaining the number of cigarettes and frequency and/or units of alcohol consumed were classified as ‘improved or continues the same’.

The way the Brazilian regions were categorized was chosen taking into consideration that most of the sample came from the Southeast region.^18^ Chronic health conditions were those reported as having been diagnosed by a physician, which included diabetes *mellitus*, hypertension, heart disease, stroke, respiratory disease, autoimmune disease, chronic kidney failure and cancer. Depression was considered separately from other chronic conditions since mental health is an important issue for the LGBT+ population. ^11^ Finally, regarding adherence to social distancing and use of masks, respondents had to fully agree that they had followed official health authority recommendations.

First, a descriptive analysis of the sample data was performed using absolute and relative frequencies. Heterogeneous differences between the categories of deteriorating lifestyle during the COVID-19 pandemic were identified by performing Pearson’s chi-square test. Logistic regression was used to estimate odds ratios (OR) and 95% confidence intervals (95%CI) in order to investigate factors associated with deteriorating lifestyle among the LGBT+ population throughout the pandemic. This analysis included only those individuals who, before the emergence of COVID-19, already had the selected lifestyles, i.e., physical activity, cigarette smoking and alcohol intake, whereby the reference category adopted was maintenance or improvement of the respective lifestyle. Separate statistical models were created for each of the outcomes. Variables with p<0.20 in the crude models were included in the adjusted models. The Hosmer-Lemeshow test was applied to analyze the fit of the final models, and the multicollinearity test was applied to test correlation between the variables included in the final models. All statistical analyses were performed using Stata 14.2 SE software (Stata-Corp., College Station, Texas, USA).

The LGBT+ Health Survey was approved on August 6^th^ 2020, by the Universidade Federal de Minas Gerais Research Ethics Committee: Certificate of Submission for Ethical Appraisal No. 34123920.9.0000.5149; Opinion No. 4.198.297. Because it was an anonymous questionnaire, a request was made for the Free and Informed Consent form to be waived. Notwithstanding, the researchers carried out the consent process, which consisted of a brief description of the research objectives and design, provided the contact details of one of the researchers in charge, and informed about potential risks and benefits for respondents.

## RESULTS

Of the 1,036 LGBT+ individuals who agreed to answer the online survey, 976 met the inclusion criteria, and of these, 975 reported having information regarding the dependent variables (regular physical activity, cigarette smoking and alcohol intake) and were included in the analysis (Figure 1).

**Figure 1:** - Final composition of the study sample after applying inclusion criteria, Brazil, August-November 2020

Between 1 and 13 data items were missing for six of the independent variables, since it was not obligatory to answer any of the questions in the survey blocks.

Regarding lifestyle deterioration during the pandemic, 48.9% (95%CI 45.7;52.1) reduced regular physical activity, 6.2% (95%CI 4.8;7.9) increased cigarette smoking, and 17.3% (95%CI 15.0;19.8) increased alcohol intake. The mean age of the participants was 31.3 years (standard deviation: 11.5).

Table 1 describes the characteristics of all respondents according to lifestyle deterioration during the pandemic. The majority were homosexual (72.2%), were of white race/skin color (60.1%), and exactly half were cisgender males (50.0%). The most frequent age group was 18 to 29 years (55.4%), and predominant schooling was up to degree level (68.4%). Higher frequency of deterioration in practicing regular physical activity was found in cisgender women, individuals in the 30-49 and 50 and over age groups, those with higher schooling (postgraduate), those who did not receive emergency government aid during the pandemic, those who did not report depression, and those who did not report a close person diagnosed - currently or previously - as having COVID-19. Greater frequency of increased cigarette smoking was found in cisgender men and transsexuals, transvestites, non-binary or other gender minorities, individuals with a single chronic health condition, and those with depression.

**Table 1:** - Characteristics of all the LGBT+ Health Survey respondents (n=975) according to lifestyle deterioration during the pandemic, Brazil, August-November 2020

Variables		Lifestyle deterioration during the pandemic			
	Total	Doing regular physical activity	Cigarette smoking	Alcohol intake
	n (%)	n (%)	n (%)	n (%)
Gender characteristics				
Affective orientation				
Homossexual	704 (72.2)	341 (48.5)	47 (6.7)	128 (18.3)
Bissexual	232 (23.8)	117 (50.4)	13 (5.7)	36 (15.6)
Other affective orientation minorities	39 (4.0)	18 (47.4)	-	4 (10.3)
Gender identity				
Cisgender male	487 (50.0)	178 (44.1)	33 (8.2)^a^	82 (20.3)
Cisgender female	405 (41.5)	259 (53.3)^a^	20 (4.1)	72 (14.9)
Transsexual, transvestite, non-binary or other gender minorities	83 (8.5)	39 (47.0)	7 (8.5)^a^	14 (16.9)
Sociodemographic characteristics				
Age group (years)				
18-29	540 (55.4)	240 (44.5)	29 (5.4)	91 (16.9)^a^
30-49	336 (34.5)	183 (54.6)^a^	25 (7.4)	66 (19.8)^a^
≥50	98 (10.1)	53 (54.1)^a^	6 (6.2)	11 (11.2)
Schooling				
Up to degree level	667 (68.4)	303 (45.5)	43 (6.5)	106 (16.0)
Postgraduate	308 (31.6)	173 (56.4)^a^	17 (5.5)	62 (20.2)
Race/skin color^b^				
Non-white	389 (39.9)	192 (49.5)	22 (5.7)	62 (16.0)
White	585 (60.1)	283 (48.5)	38 (6.5)	106 (18.2)
Living with a partner^c^				
No	718 (74.2)	343 (47.9)	41 (5.8)	113 (15.8)
Yes	250 (25.8)	128 (51.2)	19 (7.6)	53 (21.3)^a^
Number of dwellers in household^d^				
1	195 (20.2)	98 (50.3)	16 (8.2)	42 (21.8)
≥2	772 (79.8)	373 (48.4)	43 (5.6)	126 (16.4)
Region of Brazil^b^				
Southeast	781 (80.2)	384 (49.2)	49 (6.3)	131 (16.8)
Other	193 (19.8)	91 (47.6)	11 (5.8)	37 (19.3)
Receiving government emergency aid during the pandemic^e^				
No	703 (72.8)	360 (51.4)^a^	45 (6.4)	133 (19.0)^a^
Yes	262 (27.2)	111 (42.4)	15 (5.8)	35 (13.4)
Health-related characteristics				
Number of chronic health conditions^f^				
0	504 (52.4)	256 (51.0)	20 (4.0)	80 (15.9)
1	344 (35.8)	166 (48.3)	31 (9.1)^a^	68 (19.9)
≥2	114 (11.8)	46 (40.4)	9 (7.9)	19 (16.7)
Depression^f^				
No	725 (75.4)	363 (50.2)^a^	35 (4.8)	119 (16.5)
Yes	237 (24.6)	105 (44.3)	25 (10.6)^a^	48 (20.3)^a^
Someone close currently or previously diagnosed as having COVID-19^b^				
Yes	227 (23.3)	94 (41.4)	17 (7.6)	36 (15.9)
No	747 (76.7)	382 (51.3)^a^	43 (5.8)	132 (17.7)
Adherence to social distancing				
No	376 (38.6)	184 (48.9)	31 (8.3)^a^	89 (23.8)^a^
Yes	599 (61.4)	292 (48.9)	29 (4.9)	79 (13.2)
Adherence to facemask use				
No	142 (14.6)	61 (43.0)	8 (5.7)	35 (25.0)^a^
Yes	833 (85.4)	415 (49.9)	52 (6.3)^a^	133 (16.0)
N Total	975	476	60	168

a) Categories with statistically greater lifestyle deterioration frequency (p<0.05), according to Pearson’s chi-square test; missing data due to no answer being given: b) 1; c) 7; d) 12; e) 10; f) 13.

Moreover, frequency of increased cigarette smoking was also greater among those who did not adhere to social distancing and those who adhered to use of masks. Finally, greater frequency of increased alcohol intake was found among those who were younger (up to 49 years old), lived with a partner, did not receive emergency government aid during the pandemic, had depression, did not adhere to social distancing, as well as those who did not adhere to use of masks.

Table 2 shows the results of the crude and adjusted models of factors associated with each of the characteristics of lifestyle deterioration during the pandemic, considering only individuals who already had that lifestyle before the emergence of COVID-19. Since multicollinearity was not evident in any of the adjusted models (variance inflation factor <2), all independent variables with p<0.20 in the crude models were kept in the adjusted models. All models showed p>0.05 in the Hosmer-Lemeshow test, revealing adequacy. Regarding deterioration in practicing regular physical activity, statistically higher frequency was found only among those who adhered to mask use (OR=2.26; 95%CI 1.20;4.23). Among those with increased cigarette smoking, statistically higher frequency was found only among individuals with a chronic health condition (OR=2.39; 95%CI 1.03;5.56) compared to those with no chronic health condition. As for increased alcohol intake, statistically greater frequency was found in cisgender women (OR=1.95; 95%CI 1.31;2.92), compared to cisgender men, and also for the variable ‘living with a partner’ (OR=1.89; 95%CI 1.23;2.91), compared to not living with a partner. In contrast, statistically lower frequencies of increased alcohol intake were found among the ≥50 years age group (OR=0.42; 95%CI 0.19;0.89), in relation to the 18-29 age group, as well as among those receiving emergency government aid during the pandemic (OR=0.52; 95%CI 0.33;0.83), and among those adhering to social distancing (OR=0.51; 95%CI 0.35;0.75).

**Table 2:** - Crude and adjusted models of association between characteristics of the LGBT+ Health Survey respondents and lifestyle deterioration during the COVID-19 pandemic, Brazil, August-November 2020

Characteristics	Lifestyle during the pandemic							
	Reduction in regular physical activity (versus unchanged or increased) N=533			Increase in cigarette smoking (versus unchanged or increased) N=198			Increase in alcohol intake (versus unchanged or increased) N=778	
	Crude OR^a^ (95%CI^b^)	Adjusted^c^ OR (95%CI)		Crude OR (95%CI)	Adjusted^d^ OR (95%CI)		Crude OR (95%CI)	Adjusted^e^ OR (95%CI)
Gender characteristics								
Affective orientation (versus homosexual)								
Bissexual	0.91 (0.53;1.58)	-		0.78 (0.38;1.61)	-		0.81 (0.54;1.22)	-
Other affective orientation minorities	2.96 (0.37;22.58)	-		-	-		0.96 (0.31;2.94)	-
Gender identity (versus cisgender male)								
Cisgender female	0.69 (0.41;1.14)	0.64 (0.37;1.10)		1.45 (0.75;2.80)	-		1.46 (1.03;2.09)^f^	1.95 (1.31;2.92)^f^
Transsexual, transvestite, non-binary or other gender minorities	0.66 (0.28;1.52)	0.54 (0.22;1.12)		1.29 (0.46;3.59)	-		1.51 (0.78;2.90)	1.56 (0.78;3.24)
Sociodemographic characteristics								
Age group (versus 18-29 years)								
30-49 years	1.23 (0.73;2.08)	1.63 (0.85;3.11)		1.41 (0.73;2.69)	-		1.32 (0.92;1.89)	1.04 (0.66;1.63)
≥50 years	0.93 (0.44;1.97)	1.12 (0.48;2.61)		0.74 (0.27;2.01)	-		0.70 (0.35;1.37)	0.42 (0.19;0.89)^f^
Postgraduate (versus up to degree level)	0.87 (0.53;1.41)	0.70 (0.38;1.28)		1.08 (0.55;2.12)	-		1.40 (0.98;2.00)	1.06 (0.68;1.65)
Race/skin color- white (versus non-white)	0.66 (0.39;1.10)	-		1.19 (0.64;2.22)	-		1.12 (0.79;1.59)	-
Living with a partner (versus not living with a partner)	1.71 (0.92;3.15)	-		1.70 (0.86;3.35)	1.90 (0.91;3.93)		1.59 (1.09;2.31)^f^	1.89 (1.23;2.91)^f^
Number of dwellers in household ≥2 (versus 1)	1.49 (0.87;2.57)	1.61 (0.89;2.91)		0.69 (0.34;1.39)	-		0.72 (0.48;1.07)	0.65 (0.41;1.04)
Southeast region (versus other region)	0.84 (0.45;1.60)	-		0.85 (0.37;1.83)	-		0.82 (0.54;1.24)	0.85 (0.54;1.32)
Receiving government emergency aid during the pandemic (versus not receiving)	1.52 (0.81;2.86)	1.38 (0.70;2.71)		0.59 (0.30;1.16)	0.55 (0.27;1.13)		0.63 (0.42;0.95)^f^	0.52 (0.33;0.83)^f^
Health-related characteristics								
Number of chronic health conditions (versus none)								
1	1.33 (0.76;2.26)	1.45 (0.84;2.52)		2.61 (1.33;5.11)^f^	2.39 (1.03;5.56)^f^		1.36 (0.94;1.96)	1.32 (0.84;2.09)
≥2	1.69 (0.64;4.47)	1.69 (0.62;4.59)		1.39 (0.56;3.45)	0.93 (0.28;3.05)		1.33 (0.75;2.35)	1.35 (0.64;2.85)
Depression (versus not depressed)	1.52 (0.79;2.92)	-		1.89 (1.01;3.55)^f^	1.27 (0.53;3.07)		1.43 (0.97;2.10)	1.14 (0.67;1.95)
Someone close currently or previously diagnosed as having COVID-19 (versus no one)	1.31 (0.74;2.30)	-		0.63 (0.31;1.26)	0.60 (0.29;1.27)		1.10 (0.73;1.66)	-
Adherence to facemask use (versus not adhering)	0.71 (0.42;1.18)	-		0.70 (0.38;1.29)	-		0.50 (0.36;0.71)^f^	0.51 (0.35;0.75)^f^
Adesão ao uso de máscara facial (*versus* não)	1.90 (1.04;3.46)^f^	2.26 (1.20;4.23)^f^		1.82 (0.78;4.22)	2.08 (0.85;5.12)		0.55 (0.35;0.85)^f^	0.64 (0.39;1.06)

a) OR: odds ratio; b) 95%CI: 95% confidence interval; Model adequacy: c) 130.9 - p=0.41; d) 51.8 - p=0.08; e) 387.0 - p=0.15; f) p-values <0.05 based on logistic regression.

## DISCUSSION

The results of this research suggest that the lifestyles of some groups of LGBT+ individuals deteriorated when they were subjected to the environment imposed by the pandemic. Deterioration in regular physical activity was found only among those who adhered to use of masks. Increased cigarette smoking, in turn, was found only among those who reported having a single chronic health condition. Regarding increased alcohol intake, the results showed higher frequencies among females and females who lived with a partner. Still with regard to increased alcohol intake, this finding was negatively associated with the 50 years and over age group, receipt of emergency government aid during the pandemic and adherence to social distancing.

The results showed that among those who practiced regular physical activity, deterioration in this behavior was greater among those who adhered to use of masks. A Brazilian study showed that older adults who maintained healthy living habits before the pandemic tended to adhere more to preventive measures related to COVID-19.^20^ However, those who practiced physical activities adhered less to use of masks, suggesting that this measure may make it difficult to continue practicing these activities due to breathing discomfort.^20^ It should be emphasized that this study did not assess changes in lifestyles. The current stage of the pandemic may, by itself, contribute to a reduction in the number of people doing regular physical and in the time spent doing it. The results found reveal that almost 50% of the LGBT+ population had reduced regular physical activity, while among the general Brazilian population in the same age group, this reduction was 18%.^7^ In 2020, Malta et al. compared the pandemic period to public holidays and vacations, these commonly being occasions on which people relax, resulting in a reduction in movements and an increase in hours spent watching television programs and browsing on the internet, thus characterizing increased sedentary behavior on these occasions. ^7^ Despite this, according to a study conducted by Guimarães Lima et al. in 2019, ^21^ walking, soccer and bodybuilding, precisely the sports that are most practiced Brazil, go against social distancing measures, since they are practiced mainly in public spaces, which promotes contact between those who practice them. According to the theory underlying the scale called Motives for Physical Activity Measure - Revised (MPAM-R), ^22^ which can be used to measure people’s motivation to engage in physical activity, interaction with groups is determinant in this sense. Considering the pandemic scenario, these collective activities have been considerably compromised, which could impact a person’s decision to reduce physical activity. 

In relation to increased alcohol intake, mental health impairment may explain such behavior, ^23^^-^^25^ either due to fear of contamination, the need for social distancing or, moreover, because of a drop in income.^26^ The results of our study pointed to a 17.3% increase in the consumption of alcoholic beverages during the pandemic, similar to that of the general Brazilian population (17.6%),^7^ although lower than that reported in April and May 2020 (prior to the pandemic becoming established throughout Brazil), especially among its LGBT+ contingent.^14^ As for tobacco use, an increase of 42% among smokers was found (data not shown), similar to other proportional data on the Brazilian LGBT+ population (49.4%)^14^ and higher than that found for the general Brazilian population (34%).%),^7^ A study conducted with Australia’s general population in April 2020, i.e. during the pandemic, found association between deteriorating lifestyle and depression, anxiety and stress symptoms, these being conditions that are mostly more prominent among women%),^25^ and may explain increased alcohol intake. %),^25^ Unlike what was reported before the pandemic, regarding Brazilian men who have sex with men and transgender persons, %),^14^ the findings of our study showed increased alcohol consumption only among women. In the case of women with minority gender identities (including cisgender, lesbian or bisexual, and non-binary women), contents posted on social networks and related to alcohol consumption shifted from a ‘party-related’ pattern to a ‘socializing and overcoming the pandemic’ pattern. %),^27^ These findings reinforce use of alcohol as an important compensatory mechanism for coping with the pandemic among this group.

Older age groups (≥50 years), as well as those who received emergency government aid during the pandemic and who adhered to social distancing, showed less susceptibility to increased alcohol intake in the period studied, possibly a consequence of the opportunity of maintaining interaction between individuals. LGBT+ Brazilians who consumed five or more units of alcohol on a single occasion (binge drinking) showed greater difficulty in adhering to social distancing, %),^14^ according to a study the result of which corroborates that of our research. In addition, greater suffering due to social distancing has been reported for younger people, %),^25^^,^ %),^28^ belonging to the so-called potentially productive group (between 15 and 59 years old).^28^ Outside of the pandemic period, these individuals are expected to have more social interaction and therefore, at a time of mandatory isolation, are expected to be the most mentally impacted by COVID-19. ^25^^,^^28^ Among the more vulnerable, especially those who received emergency government aid, although their mental health was also considerably impacted by unemployment and financial difficulty, ^5^ this was not reflected in increased alcohol intake. More vulnerable groups had to give priority to food^5^ and somewhere to live, so that less money was left over for alcohol intake.

Despite having strong points, such as being the first nationwide survey to investigate the topic and to highlight lifestyle deterioration in a historically neglected population, some limitations should be raised. First, the inherent limitations of the online survey method, such as non-representativeness and the volunteer effect, ^29^ excluding the most vulnerable population that also does not have access to the internet. Thus, selection bias cannot be ruled out and therefore the results of the study cannot be generalized to the entire LGBT+ population. Second, the response rate was not evaluated, since the software used did not allow this, in addition to duplicated information, given that it was an anonymous survey. Third, no weights were used for possible statistical corrections to increase the representativeness of the target population, since there was a large concentration of respondents in the Southeast region. Fourth, the cross-sectional nature of the survey did not allow the direction of the associations found to be known. And finally, all outcomes were measured based on self-reporting, which may be influenced by other mental factors not included in the research, besides being subject to individual perception and information bias.

In conclusion, the results reinforce that individuals identified as belonging to the LGBT+ group showed vulnerability, regarding lifestyle deterioration during the COVID-19 pandemic. Standing out among the factors associated with lifestyle deterioration were (i) individuals who adhered to wearing masks, (ii) those with a chronic health condition, and (iii) cisgender females. These results suggest that the prolongation of the pandemic and social distancing measures may impact the rate of health deterioration among the LGBT+ population.
